# Magnetic Resonance Imaging of Ferumoxytol-Labeled Human Mesenchymal Stem Cells in the Mouse Brain

**DOI:** 10.1007/s12015-016-9694-0

**Published:** 2016-10-18

**Authors:** Na Kyung Lee, Hyeong Seop Kim, Dongkyeom Yoo, Jung Won Hwang, Soo Jin Choi, Wonil Oh, Jong Wook Chang, Duk L. Na

**Affiliations:** 1 Department of Health Sciences and Technology, SAIHST, Sungkyunkwan University, 135-710 Seoul, Republic of Korea; 2 Department of Neurology, Samsung Medical Center, Sungkyunkwan University School of Medicine, 81 Irwon-dong, Gangnam-gu, Seoul, 135-710 Republic of Korea; 3 Neuroscience Center, Samsung Medical Center, Seoul, Republic of Korea; 4 Stem Cell & Regenerative Medicine Institute, Samsung Medical Center, 135-710 Seoul, Republic of Korea; 5 Center for Molecular & Cellular Imaging, Samsung Biomedical Research Institute, Seoul, Republic of Korea; 6 Biomedical Research Institute, MEDIPOST Co., Ltd., 463-400 Gyeonggi-do, Republic of Korea

**Keywords:** Mesenchymal stem cells, Ferumoxytol, Monitoring, Magnetic resonance imaging, Stem cell therapy

## Abstract

The success of stem cell therapy is highly dependent on accurate delivery of stem cells to the target site of interest. Possible ways to track the distribution of MSCs in vivo include the use of reporter genes or nanoparticles. The U.S. Food and Drug Administration (FDA) has approved ferumoxytol (Feraheme® [USA], Rienso® [UK]) as a treatment for iron deficiency anemia. Ferumoxytol is an ultrasmall superparamagnetic iron oxide nanoparticle (USPIO) that has recently been used to track the fate of transplanted cells using magnetic resonance imaging (MRI). The major objectives of this study were to demonstrate the feasibility of labeling hUCB-MSCs with ferumoxytol and to observe, through MRI, the engraftment of ferumoxytol-labeled human umbilical cord blood-derived mesenchymal stem cells (hUCB-MSCs) delivered via stereotactic injection into the hippocampi of a transgenic mouse model of familial Alzheimer’s disease (5XFAD). Ferumoxytol had no toxic effects on the viability or stemness of hUCB-MSCs when assessed *in vitro*. Through MRI, hypointense signals were discernible at the site where ferumoxytol-labeled human MSCs were injected. Iron-positive areas were also observed in the engrafted hippocampi. The results from this study support the use of nanoparticle labeling to monitor transplanted MSCs in real time as a follow-up for AD stem cell therapy in the clinical field.

**Electronic supplementary material:**

The online version of this article (doi:10.1007/s12015-016-9694-0) contains supplementary material, which is available to authorized users.

## Introduction

The curative and regenerative properties of stem cells have marked them as promising candidates as novel therapeutic agents for various neurodegenerative diseases [1–3]. Out of the wide range of neurodegenerative diseases, stem cell application seems to be particularly appealing for Alzheimer’s disease (AD) [4–6]. Considered the most widely known and common type of dementia, the pathology of AD is complex and the causes are multifactorial [7, 8]. With such complications present, mesenchymal stem cells (MSCs) have become a promising therapeutic agent for AD stem cell therapy because of the multiple functional roles that they can mediate through their paracrine activities [9–11]. The multiple roles of MSCs, ranging from anti-apoptosis to anti-inflammation, make them ideal for the treatment of heterogeneous diseases such as AD where they can target more than one mechanism [12–15]. Using a transgenic AD mouse model, our group has observed the removal of amyloid plaques and acceleration of endogenous neurogenesis through treatment with MSCs [16–18].

In order for these MSCs to exert their therapeutic benefits at the target site, the delivery route is of key concern [19]. Previously, our group has reported that MSC engraftment in the brains of transgenic AD mice is very low when cells are delivered through the intra-venous [20] and intra-arterial routes [21]. In contrast, intra-parenchymal administration is invasive, although maximal engraftment of MSCs can be achieved. In parallel to our pre-clinical studies [16, 17, 20], our group has also conducted a phase I clinical trial involving the intra-parenchymal administration of human umbilical cord blood-derived mesenchymal stem cells (hUCB-MSCs) into the hippocampus and precuneus of AD patients [22]. Through this study, we were able to confirm the safety and feasibility of implanting cells directly into the brain parenchyma. However, one limitation was that the distribution of the transplanted cells could not be assessed through non-invasive means.

Cells can be observed non-invasively using two methods: a direct method that involves labeling stem cells with nanoparticles and an indirect imaging method that utilizes reporter genes [23, 24]. Out of the various imaging modalities, magnetic resonance imaging (MRI) possesses several advantages that make it suitable for cellular imaging, such as superior soft tissue contrast and high resolution [25]. Cells can be labeled with iron oxide nanoparticles to perform MRI [26, 27]. In 2009, the U.S. Food and Drug Administration (FDA) approved the use of ferumoxytol (Feraheme® [AMAG Pharmaceuticals, USA], Rienso® [Takeda Inc., UK]), an ultrasmall superparamagnetic iron oxide nanoparticle (USPIO), as a treatment for iron deficiency anemia in chronic kidney disease [28, 29]. Ferumoxytol (off-label use) has been used widely in pre-clinical studies for cellular imaging purposes [29–32].

Utilizing these characteristics of ferumoxytol, the major objectives of this current study were first to investigate the feasibility of labeling hUCB-MSCs with ferumoxytol *in vitro,* and then to monitor in vivo engraftment of MSCs through MRI. To reduce the risk of engraftment failure, intra-parenchymal administration was chosen to non-invasively image MSCs that had been directly delivered into the hippocampus of the 5X familial Alzheimer’s disease (FAD) mouse model.

## Materials and Methods

### Ethical Statement

This study was reviewed and approved by the Institutional Review Board (IRB) of Samsung Medical Center (IRB no. 2015–01-028). This research was also approved by the Institutional Animal Care and Use Committee (IACUC) of the Samsung Biomedical Research Institute (SBRI) at Samsung Medical Center (SMC). The SBRI is an accredited facility of the Association for Assessment and Accreditation of Laboratory Animal Care International (AAALAC International) and abides by the Institute of Laboratory Animal Resources (ILAR) guide.

### Cell Culture and Ferumoxytol Labeling

Human umbilical cord blood-derived mesenchymal stem cells (hUCB-MSCs) acquired from Medipost Inc. (Biomedical Research Institute Co., Ltd., Republic of Korea) were cultured in minimum essential medium (MEM)α1× media (Gibco-Invitrogen, Carlsbad, CA, USA) containing 10 % fetal bovine serum (FBS; Biowest, Riverside, MO, USA) and 0.5 % gentamicin (Thermo Fisher Scientific, Hudson, NH, USA) at 37 °C, 5 % CO_2_. Passage 6 cells were used for the study.

After reaching 80–90 % confluence, hUCB-MSCs were washed with Dulbecco’s phosphate buffered saline (DPBS; Biowest, Riverside, MO, USA) and labeled with ferumoxytol as described previously [29, 33, 34]. Cells were treated with serum-free MEMα1× medium containing heparin (4 U/mL; JW Pharmaceuticals, Seoul, Republic of Korea), protamine sulfate (80 μg/mL; Hanlim Pharmaceuticals, Republic of Korea), and ferumoxytol (200 μg/mL; Rienso®, Takeda Inc., Denmark, UK). These reagents are clinically available and thus readily accessible for use. After 4 to 5 h, an equal volume of medium supplemented with 20 % FBS was added to give a final concentration of 2 U/mL heparin, 40 μg/mL protamine sulfate, and 100 μg/mL ferumoxytol. Cells were incubated for an additional 20 h at 37 °C, 5 % CO_2_.

### Cell Viability Assay

hUCB-MSCs were initially seeded in six replicates of 96-well plates at a density of 9.6 × 10^3^ per well for 24 h. MSCs were treated with 2 U/mL heparin and various doses of protamine sulfate and ferumoxytol for an additional 24 h. After the incubation period, cells were assayed for viability using the Alamar blue assay (Sigma-Aldrich, St. Louis, MO, USA). Cells were treated with the Alamar blue reagent for 3 h at 37 °C and 5 % CO_2_, and fluorescence was read by a multiplate reader (GloMax®-Multi Detection System; Promega, Madison, WI, USA).

### Prussian Blue Staining

Unlabeled and ferumoxytol-labeled hUCB-MSCs were washed with DPBS (Biowest) and then fixed with 4 % paraformaldehyde (Biosesang, Gyeonggi-do, Republic of Korea) for 15 min at room temperature (RT). Cells were washed with DPBS before staining. Paraffin blocks of the ferumoxytol-labeled hUCB-MSCs were prepared as described previously [21]. Staining was performed as instructed by the manufacturer (NovaUltra Prussian Blue Stain Kit; IHC WORLD, Woodstock, MD, USA). Stained slides were scanned using Aperio Scan Scope AT and visualized through the Aperio Image Scope program (Leica Biosystems, Buffalo Grove, IL, USA).

### Immunophenotyping

After 24 h, unlabeled and ferumoxytol-labeled hUCB-MSCs were washed with DPBS and detached using 0.25 % trypsin (Sigma-Aldrich). The surface antigens of unlabeled and ferumoxytol-labeled hUCB-MSCs were phenotyped by staining the cells with FITC, PE, or APC-coupled antibodies for 15 min at RT. Anti-human antibodies against the following proteins were used for fluorescence-activated cell sorting (FACS): CD14, CD45, CD73, CD90, CD105, and HLA-DR (BD Pharmingen, San Jose, CA, USA). IgG1 and IgG2a (BD Pharmingen) were used as the corresponding mouse isotype controls. Labeled cells were washed with DPBS, fixed with 1 % paraformaldehyde (PFA; Biosesang, Gyeonggi-do, Republic of Korea), and analyzed by the MACSQuant® Analyzer (Miltenyi Biotec, San Diego, CA, USA).

### Trilineage Differentiation and Evaluation

Adipogenic differentiation was induced using the StemPro Adipogenesis Differentiation Kit (Thermo Fisher Scientific). hUCB-MSCs were labeled with ferumoxytol for 24 h in a 6-well plate, washed three times with DPBS, and the media was replaced with the adipogenic base medium. The medium was changed twice a week for a total of 2 weeks. Cells were fixed with 4 % PFA and stained with Oil Red O (Sigma-Aldrich). To induce osteogenic differentiation, cells were first labeled with ferumoxytol as described above and then cultured in osteogenic base medium using the StemPro Osteogenesis Differentiation Kit (Thermo Fisher Scientific). The medium was changed twice a week for one week. After fixation using a solution containing citrate and acetone, mineralized matrix was assessed by alkaline phosphatase staining (Sigma-Aldrich).

Unlabeled and ferumoxytol-labeled cells were treated with chondrogenic medium, which consisted of high-glucose DMEM (Biowest) supplemented with 100 nM dexamethasone (Sigma-Aldrich), 50 mg/mL L-ascorbic acid (Sigma-Aldrich), 100 mg/mL sodium pyruvate (Sigma-Aldrich), 40 mg/mL L-proline (Sigma-Aldrich), 10 ng/mL transforming growth factor β3 (TGF-β3; R&D Systems, Minneapolis, MN, USA), 500 ng/mL bone morphogenic protein 6 (BMP-6; R&D Systems), and 50 mg/mL ITS+ premix (Becton Dickinson, Franklin Lakes, NJ, USA). After induction of differentiation for 4 weeks, cell pellets were collected and embedded in OCT compound (Tissue-Tek, Torrance, CA, USA). Sections of the pellets were prepared at 5-μm thickness using a cryotome (Thermo Fisher Scientific) and stained with Safranin-O (Biosesang). Stained slides were observed using an inverted light microscope (U-HGLGPS, Olympus, Japan).

### Experimental Animals

5XFAD transgenic AD mice that overexpress mutant human APP(695) with the Swedish (K670 N, M671 L), Florida (I716V), and London (V717I) familial Alzheimer’s disease (FAD) mutations and human PS1 harboring two FAD mutations, M146 L and L286 V, were purchased from Jackson Laboratories (Bar Harbor, ME, USA). These transgenic mice were crossed, and the offspring were genotyped by DNA extraction from tail samples. These mice were fed ad libitum and were also maintained in a 12 h light/12 h dark cycle. A total of fifteen 7–8 month old mice were used for this study: sham (n = 3), MR imaging right after injection (n = 3), post 1 day (n = 3), 7 days (n = 3), and 14 days (n = 3).

### Hippocampal Injections of Unlabeled and Ferumoxytol-Labeled hUCB-MSCs

After labeling with ferumoxytol for 24 h, hUCB-MSCs were washed three times: first with DPBS, second with DPBS containing heparin (10 U/mL), and finally with DPBS again. Cells were detached using 0.25 % Trypsin-EDTA (Gibco-Invitrogen). Ferumoxytol-labeled hUCB-MSCs were resuspended in phenol red-free MEMα1× media (Gibco-Invitrogen) prior to injection. Animals were stably positioned on a stereotactic device (Harvard Apparatus, Holliston, MA, USA) using ear bars. Coordinates of A/P − 1.94 mm, M/L ± 1.00 mm, and D/V − 2.6 mm were used to perform the sham operation, which only involved insertion and removal of a Hamilton syringe needle (Hamilton Company, Reno, NV) or injection of unlabeled (2 × 10^5^ /3 μL), and/or ferumoxytol-labeled hUCB-MSCs (2 × 10^5^/3 μL) into the left and right hippocampus of 5XFAD mice, respectively, at a rate of 0.5 μl/min. Following the infusion, a 5 min delay time was provided before retracting the syringe needle. Mice were initially anesthetized with 5 % isoflurane (Hana Pharmaceutical Co., Ltd., Seoul, Republic of Korea) and subsequently maintained under 2 % isoflurane during the whole procedure.

### Magnetic Resonance Imaging

A 7 T/20 MRI System (Bruker-Biospin, Fällanden, Switzerland) was used to acquire MR images of the phantom cell samples and the mouse brain. Unlabeled and ferumoxytol-labeled cells were mixed with 0.8 % agarose (Bioplus, Gyeonggi-do, Republic of Korea) to prepare the phantom samples. The tubes were filled with distilled water to reduce background artifacts. MR images were obtained using a multislice multiecho (MSME) spin echo sequence with the following parameters: repetition time (TR)/echo time (TE) = 2500/14–230 msec, slice thickness = 0.7 mm, number of averages = 4. MR images of the mouse brain were acquired as described previously [﻿ 21]. T2-weighted images were obtained using the following parameters: TR/TE = 2500/20 msec, number of averages = 8, and slice thickness = 0.7 mm. A T2* gradient echo (GRE) sequence was used to acquire MR images using the following parameters: TR/TE = 303.805/18 msec, number of averages =8, slice thickness = 0.7 mm, and flip angle (FA) = 45°. Mice were anesthetized under 2 % isoflurane while the MR images were obtained.

### Tissue Fixation and Immunohistochemistry

At the respective time points, mice underwent MR imaging and were sacrificed via cardiac perfusion. After fixation in 4 % PFA for 24 h, paraffin blocks were prepared, and 4-μm sections were cut using a microtome (Thermo Fisher Scientific). Sections were deparaffinized, and Prussian blue staining was conducted using the protocol described above. For immunohistochemical (IHC) staining, 1× citrate buffer, pH 6 (Dako, Carpinteria, CA, USA) was first used to perform heat-induced antigen retrieval. Immunofluorescence staining was performed according to our previous report [21] using primary antibodies against mitochondria (1:200; Millipore, Billerica, MA, USA), ionized calcium binding adaptor molecule 1 (Iba-1, 1:250; Wako Chemicals, Richmond, VA, USA), and glial fibrillary acidic protein (GFAP, 1:1000; Abcam, Cambridge, MA, USA). Alexa Fluor 546-conjugated donkey anti-mouse (1:250; Life Technologies, Hudson, NH, USA), Alexa Fluor 488-conjugated donkey anti-goat (1:400; Jackson ImmunoResearch Europe Ltd., Newmarket, UK), and Alexa Fluor 546-conjugated donkey anti-rabbit (1:400; Life Technologies, Hudson, NH, USA) were used as secondary antibodies. Stained slides were observed using a confocal microscope (LSM700, Carl Zeiss AG, Jena, Germany).

### Statistical Analysis

All data presented in the paper are expressed as mean ± standard error of mean (S.E.M.). A *P* value ≤0.05 was considered statistically significant. Student’s t-test was used to investigate the differences between groups.

## Results

### Characterization of Ferumoxytol-Labeled hUCB-MSCs *in vitro*

**Fig. 1:**
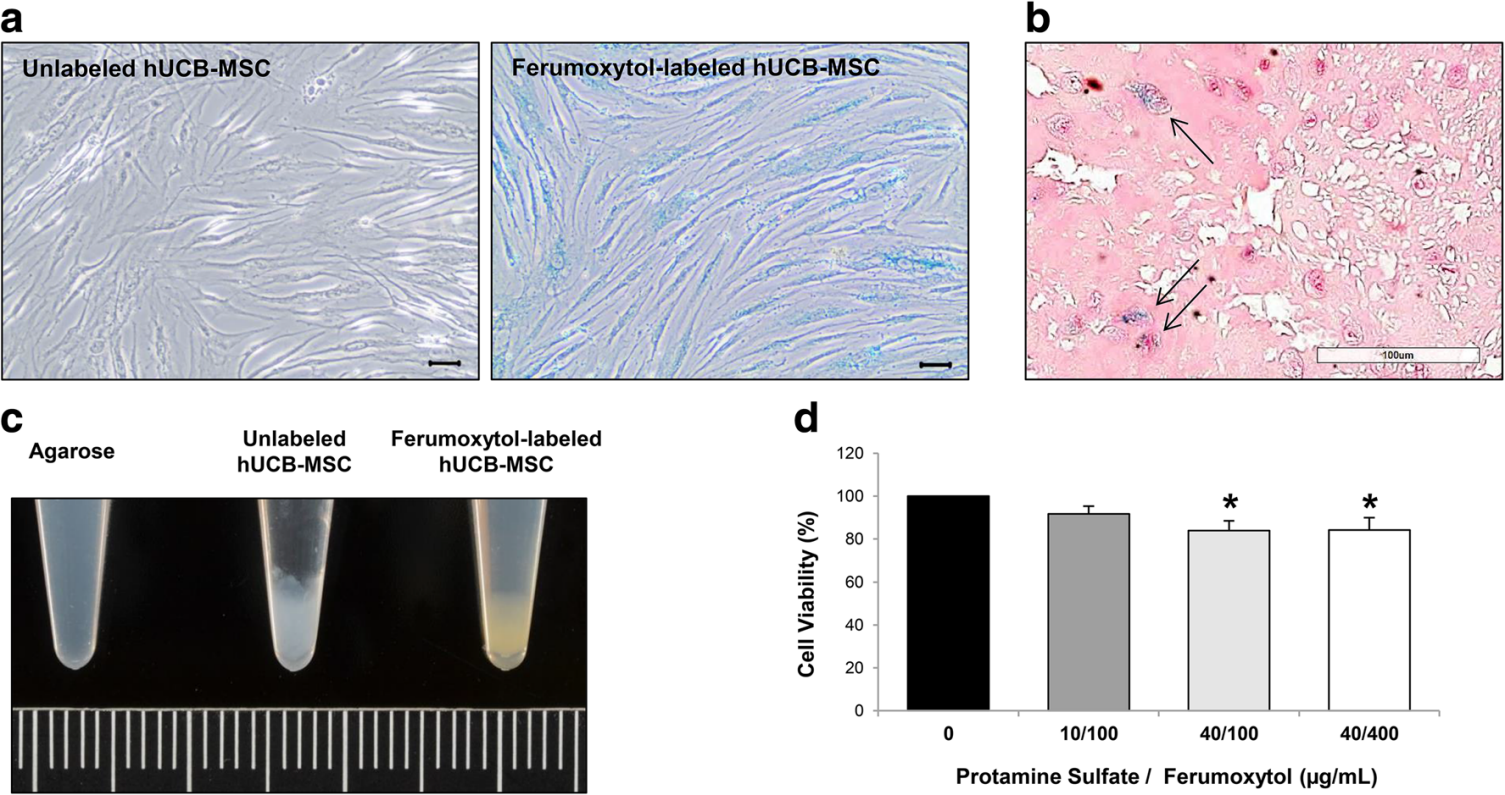
Labeling hUCB-MSCs with Ferumoxytol. **a** Compared to unlabeled cells where no iron-positive blue areas were evident, blue staining could be observed in the cytoplasm of ferumoxytol–labeled hUCB-MSCs. Scale bar =50 μm. **b** Co-localization of iron- (black solid arrow) and ferumoxytol-labeled hUCB-MSCs (circular, pink) in a re-suspended state. Scale bar =100 μm. **c** Brown staining could be detected from the pellet of ferumoxytol-labeled hUCB-MSCs. Grid =1 mm. **d** Cell viability of hUCB-MSCs treated with various concentrations of protamine sulfate/ferumoxytol assessed using the Alamar Blue assay (average of 4 independent experiments, *P ≤ 0.05 compared to the unlabeled control sample)

Using the heparin-protamine sulfate-ferumoxytol nanocomplex technique, blue staining was seen in the cytoplasm of hUCB-MSCs labeled with ferumoxytol compared with the unlabeled hUCB-MSCs (Fig. 1a). Differences in morphology of hUCB-MSCs such as adherent, bipolar, and fibroblast-like characteristics [35] were not noted following labeling (Fig. 1a). The efficiency of ferumoxytol labeling of hUCB-MSCs was further observed in the detached re-suspended state by creating cell paraffin blocks (Fig. 1b). Unlike the unlabeled hUCB-MSCs, brown staining was grossly observed from the pellet of ferumoxytol-labeled hUCB-MSCs (Fig. 1c), a common observation for cells labeled with iron oxide nanoparticles [36, 37]. The overall labeling efficiency was 85.84 ± 2.02 % (mean ± S.E.M.). To assess alterations in cell viability, hUCB-MSCs were treated with a constant dose of heparin and varying doses of protamine sulfate and ferumoxytol. The cell viability percentages after treatment with 10/100, 40/100, and 40/400 μg/mL of protamine sulfate/ferumoxytol were 91.64 ± 3.66, 83.86 ± 4.58, and 84.19 ± 5.76 %, respectively (Fig. 1d). Labeling the cells did not affect the overall viability of the cells; however, increasing the concentration of protamine sulfate decreased the viability by approximately 20 %. A possible explanation is the toxic effects exerted by the positive charges of protamine sulfate [38]. However, the overall viability was still high probably due to the counteractive roles of heparin on protamine sulfate [38].

According to the axial and sagittal MR images, of the agarose-only, unlabeled, and ferumoxytol-labeled hUCB-MSC-agarose phantom samples, a reduction in signal contrast was detected from ferumoxytol-labeled hUCB-MSCs (Supplementary Fig. S1A). When quantitatively measured, the mean signal intensities of the agarose sample, unlabeled, and ferumoxytol-labeled hUCB-MSCs were 168.33 ± 4.48, 151 ± 2.08, and 63.67 ± 6.34, respectively (Supplementary Fig. S1B). This reduction in signal intensity was statistically significant when the ferumoxytol-labeled hUCB-MSCs were separately compared with the agarose control (**P ≤ 0.01) and the unlabeled hUCB-MSCs (**P ≤ 0.01) (Supplementary Fig. S1B). The difference between the unlabeled hUCB-MSCs and the agarose-only sample was also statistically significant (*P ≤ 0.05).

### Differentiation Potential and Stemness of Ferumoxytol-Labeled hUCB-MSCs

**Fig. 2:**
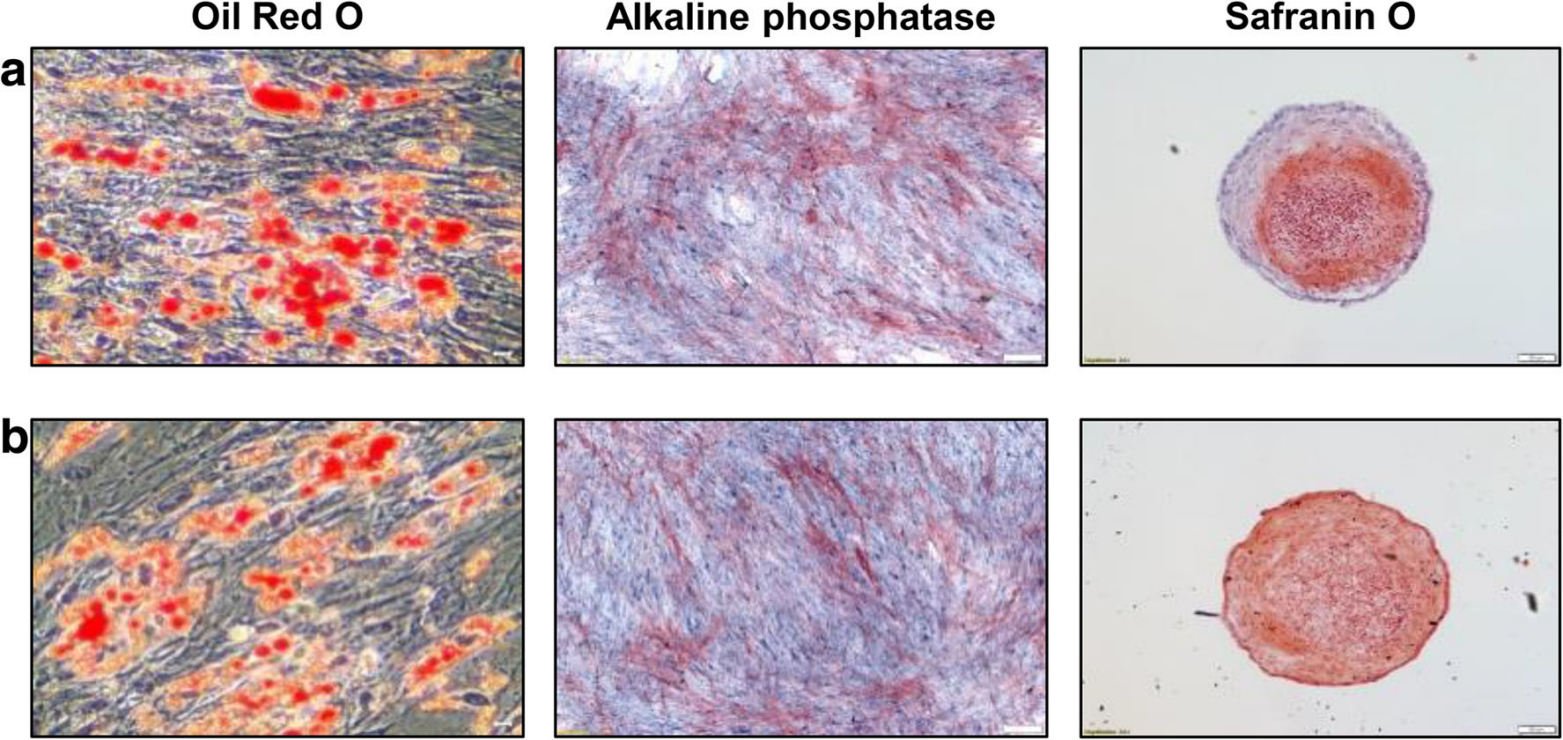
Differentiation Potential of Ferumoxytol-Labeled hUCB-MSCs. Adipogenic (Oil Red O staining), osteogenic (alkaline phosphatase), and chondrogenic (Safranin O staining) differentiation were observed from both (**a**) unlabeled and (**b**) ferumoxytol-labeled hUCB-MSCs. Oil Red O: Scale bar =50 μm; Alkaline phosphatase and Safranin O = 100 μm

Trilineage differentiation was not affected by labeling hUCB-MSCs with ferumoxytol. For both unlabeled (Fig. 2a) and ferumoxytol-labeled hUCB-MSCs (Fig. 2b), induction of adipogenesis was demonstrated by the accumulation of lipid-rich vacuoles inside the cells that were stained with Oil red O (Fig. 2a, b). Osteogenic differentiation was observed in both unlabeled and ferumoxytol-labeled cells that expressed alkaline phosphatase (Fig. 2a, b). The expression of sulfated proteoglycans was detected by Safranin O staining (Fig. 2 a, b).

Through FACS, there were no discernible differences in the expression of surface molecules between the unlabeled and ferumoxytol-labeled hUCB-MSCs. The percentages of positive expression of surface molecules CD105, CD90, and CD73 were 99.78 ± 0.09, 99.06 ± 0.44, and 99.92 ± 0.03 %, respectively, for unlabeled hUCB-MSCs (Supplementary Fig. S2A) and 99.98 ± 0.01, 98.43 ± 0.73, and 99.91 ± 0.07 %, respectively, for ferumoxytol-labeled hUCB-MSCs (Supplementary Fig. S2B). Both unlabeled and ferumoxytol-labeled hUCB-MSCs lacked expression of the surface molecules HLA-DR, CD45, and CD14 (Supplementary Fig. S2); the expression percentages were ≤2 %, which met the minimal criteria proposed by the International Society for Cellular Therapy (ISCT) for MSCs [35, 39].

### Detection of Hypointense Signals from Transplantation of Ferumoxytol-Labeled hUCB-MSCs in the Mouse Hippocampus

**Fig. 3:**
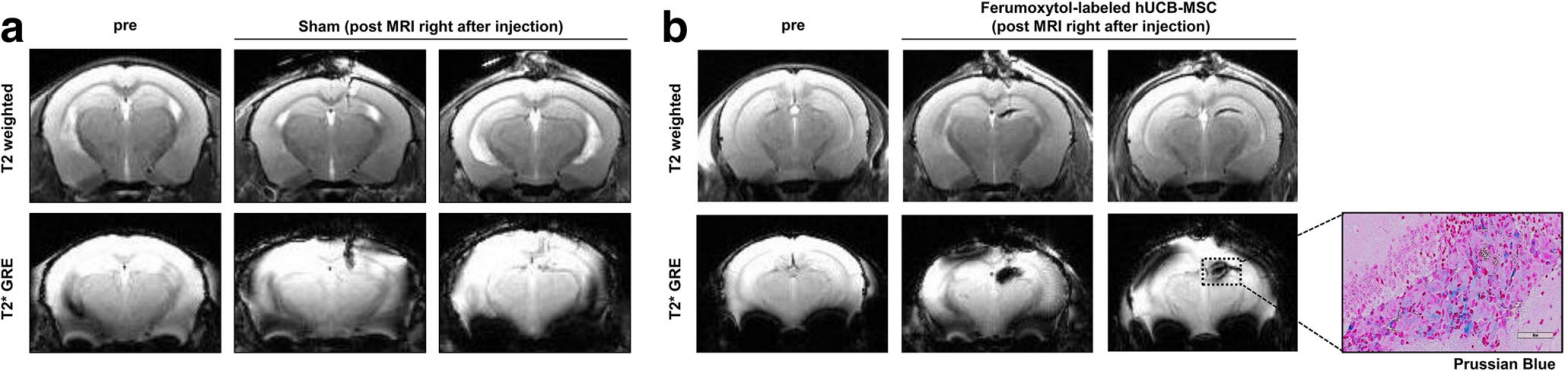
Differences in Hypointensities Generated from Sham-Operated and Ferumoxytol-labeled hUCB-MSC Injected Mice. **a** A linear hypointense signal from T2-weighted and T2* GRE images revealed the puncture site and needle track. **b** In contrast, diffusion of hypointense signals was discernible in the right hippocampus of mice that received injections of ferumoxytol-labeled hUCB-MSCs. Prussian blue stains revealed iron positive cells in the right hippocampus. Scale bar: 80 μm

The in vivo MR characteristics of ferumoxytol-labeled hUCB-MSCs were preliminarily assessed by performing a sham surgery and intra-parenchymal administration of ferumoxytol-labeled MSCs into the right hippocampus. MR images were acquired immediately after surgery. In the T2-weighted and T2* GRE MR images, the sham group displayed a hypointense (dark) vertical line extending downward from the cortex, representing the location of the needle track (Fig. 3a). However, the manifestation of the hypointense signal was clearly distinguishable from the group that received ferumoxytol-labeled hUCB-MSC injections, which showed hypointense signals that permeated or spread at the end of the needle track and created a diagonal linear (T2-weighted) or ovular (T2* GRE) appearance (Fig. 3b). The presence of ferumoxytol-labeled hUCB-MSCs was confirmed through Prussian blue staining (Fig. 3b).

**Fig. 4:**
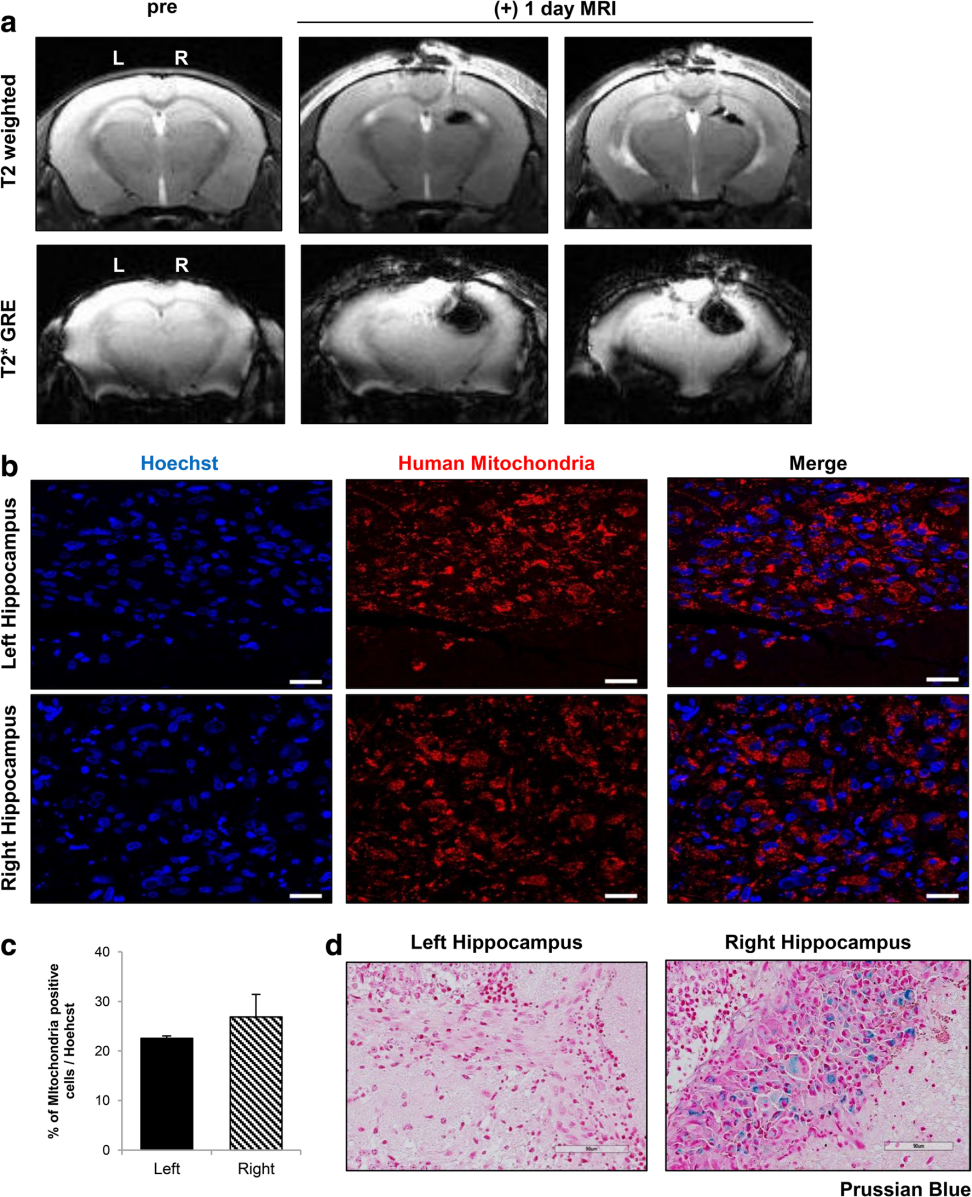
Post 1 Day MR Images Corroborated with IHC Staining. **a** A total of 2 × 10^5^ unlabeled or ferumoxytol labeled hUCB-MSCs were injected into the left and right hippocampus, respectively, of 5XFAD mice. According to the T2-weighted and T2* MR images acquired post 1 day, compared to the left hemisphere, hypointense signals could be observed from the right hippocampus where ferumoxytol-labeled hUCB-MSCs were administered. Left (L) = unlabeled hUCB-MSCs, and right (R) = ferumoxytol-labeled hUCB-MSCs. **b** Human MSCs were observed in both the left and right hippocampi using the anti-mitochondria antibody. Scale bar: 20 μm. **c** Quantification of the number of cells positive for anti-mitochondria staining over the total number of Hoechst-positive cells (4 sections per hippocampus for each mouse). **d** Iron deposits were detected by Prussian blue staining in the right hippocampus. Scale bar: 90 μm

Subsequently, engraftment of ferumoxytol-labeled hUCB-MSCs was observed at post 1 day. MR images acquired at 1 day post injection displayed dark, hypointense signals in the right hippocampus of 5XFAD mice where the ferumoxytol-labeled hUCB-MSCs were injected (Fig. 4a). Although a strong blooming effect was evident, the appearance of the signal was similar to that from MR images acquired immediately after the transplantation. Other than slight hyperintense signals showing the site of needle puncture in the T2-weighted MR images, no hypointense signals were detected in the left hippocampus, where unlabeled hUCB-MSCs were administered (Fig.4a).

**Fig. 5:**
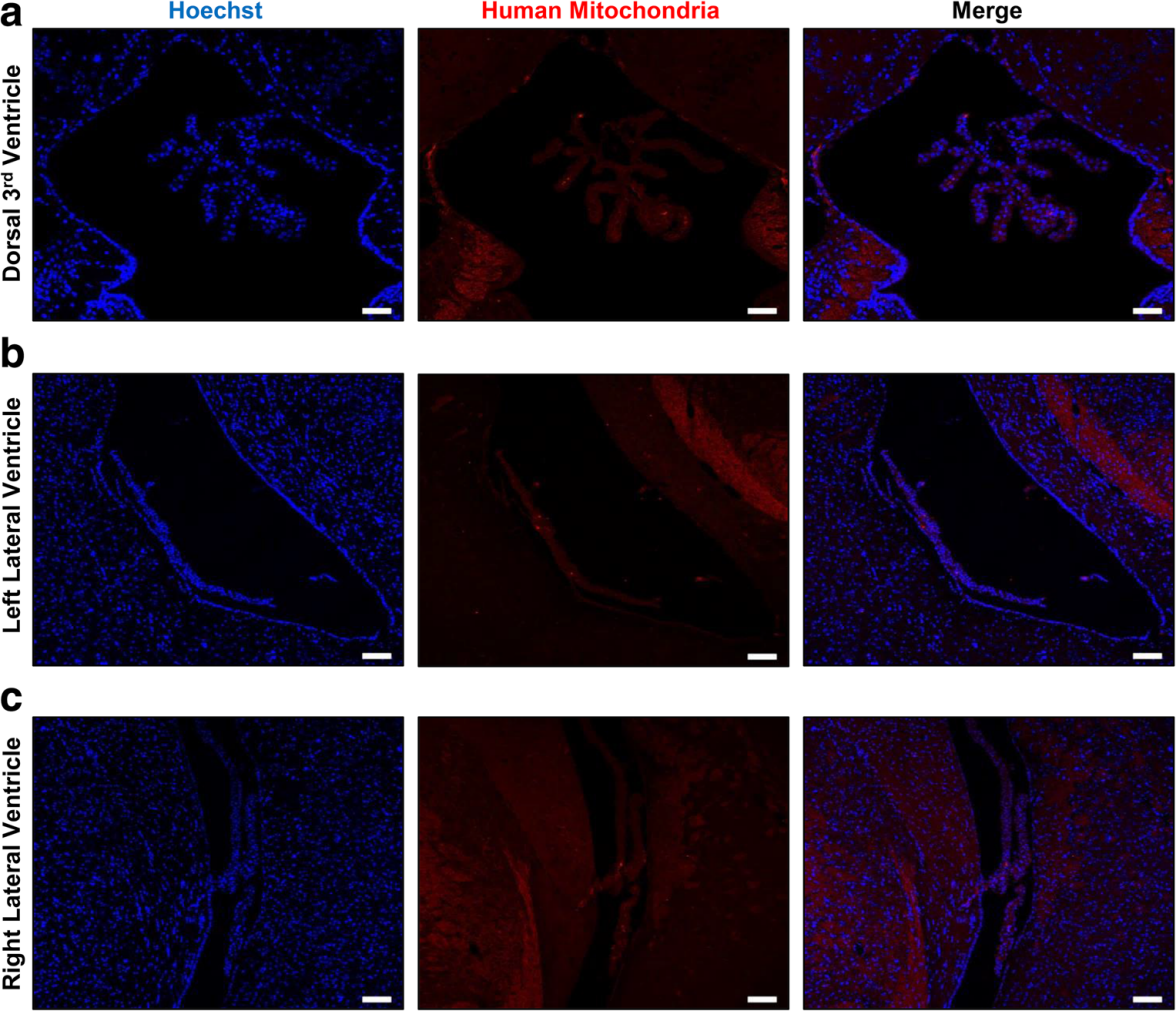
Absence of MSCs in the Vascular Regions of the Mouse Brain. No hUCB-MSCs (anti-mitochondria antibody) were discernible in the (**a**) dorsal 3rd ventricle or in either the (**b**) left or (**c**) right lateral ventricle. Scale bar: dorsal 3rd ventricle: 50 μm, lateral ventricles: 100 μm

Human MSC engraftment in both hippocampi (Fig. 4a) was confirmed by IHC staining of human mitochondria (Fig. 4b). Although the percentage of mitochondria-positive cells among the total cells (Hoechst stained) was slightly higher in the right hemisphere, the differences between the two hippocampi were not statistically significant (Fig. 4c). In addition to corroborating human MSC engraftment, iron-positive areas were stained blue in the right hippocampus by Prussian blue staining, whereas none were discernible in the left hippocampus (Fig. 4d). The absence of human cells in the dorsal 3rd ventricle (Fig. 5a) and lateral ventricles of the left and right hemispheres (Fig. 5b, c) confirmed the accuracy of the engraftment and suggested that the cells did not migrate into ventricular regions by possible back flow that may arise along the needle track.

### No Infiltration of Inflammatory Cells into the Site of MSC Engraftment

**Fig. 6:**
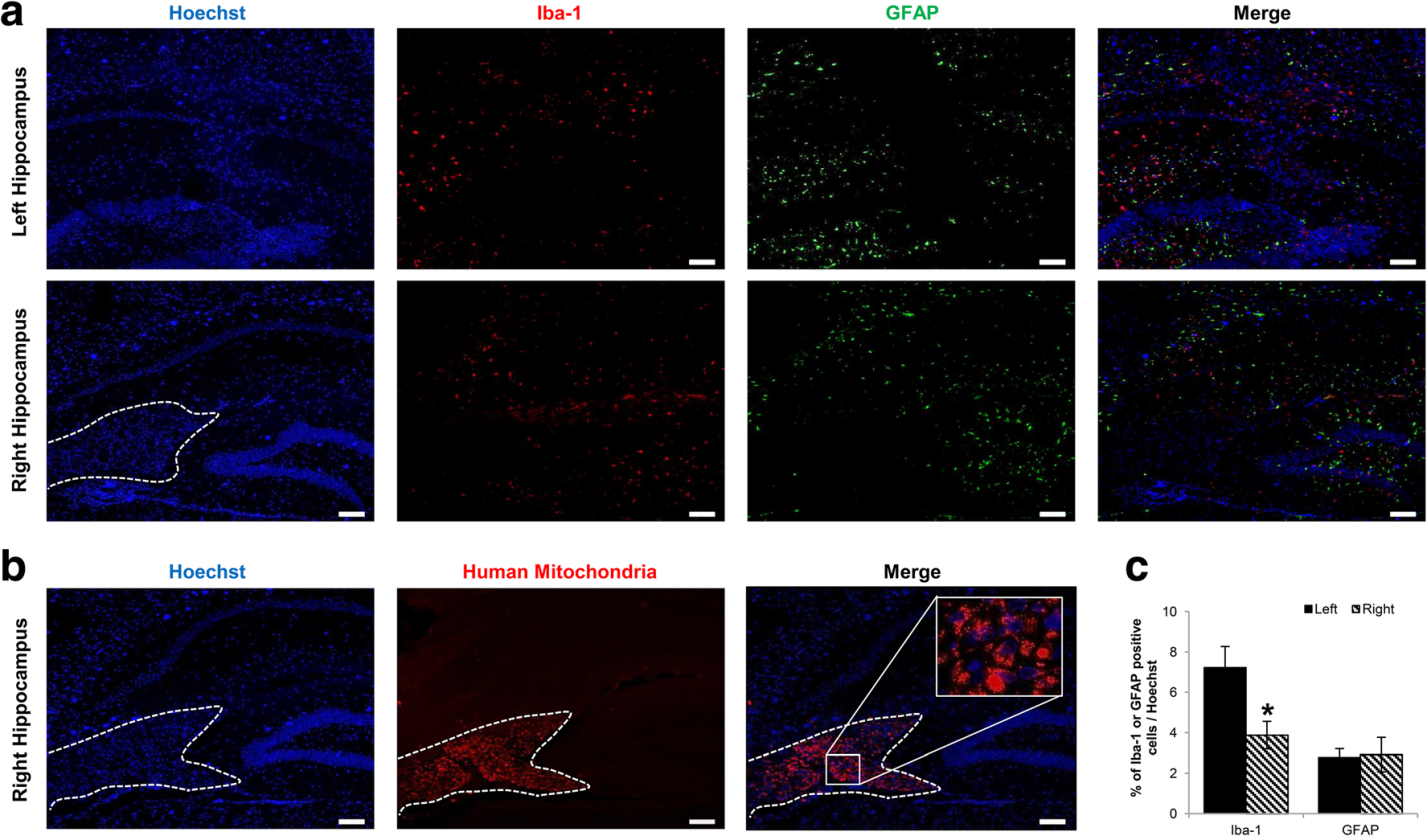
Inflammatory Cells are not Observed at the Sites of hUCB-MSC Engraftment. **a** Iba-1 and GFAP expression was not observed at the locations where hUCB-MSCs were transplanted in either the left or right hippocampus. The white dotted line indicates the site of cell engraftment. Scale bar: 100 μm. **b** An adjacent slide was stained using the anti-mitochondria antibody to confirm the location of the cells enclosed by the white dotted line. (C) Quantification of number of cells positive for Iba-1 (left) or GFAP (right) over total number of Hoechst-positive cells (4 sections per hippocampus for each mouse)

Additional IHC staining using anti-Iba-1 (microglia/macrophage) and anti-GFAP (astrocyte) antibodies was performed to evaluate inflammatory effects that may arise from injection of unlabeled/ferumoxytol-labeled hUCB-MSCs and to corroborate the source of the MR signal. Based on microscopic evaluations, a dramatic difference in Iba-1 and GFAP expression was not evident between the left and right hippocampus (Fig. 6a). Interestingly, very low or no expression of Iba-1 or GFAP was apparent at the site of hUCB-MSC engraftment (indicated with a white dotted line) in the right hippocampus, which was confirmed by staining an adjacent slide using the anti-mitochondria antibody (Fig. 6b). These observations were quantitatively assessed, and the percentages of Iba-1 and GFAP–positive cells over the total number of cells (Hoechst) were determined for both the left and right hippocampi (Fig. 6c). Compared to the left hemisphere, the percentages of Iba-1 and GFAP were lower or similar, respectively, in the right hippocampus. Although not investigated in detail, the slightly higher engraftment percentage of MSCs in the right hippocampus could have exerted more immunomodulatory effects to contribute to the lower Iba-1 percentage in comparison to the left hippocampus.

### Persistence of Hypointense Signals at Prolonged Time Points

**Fig. 7:**
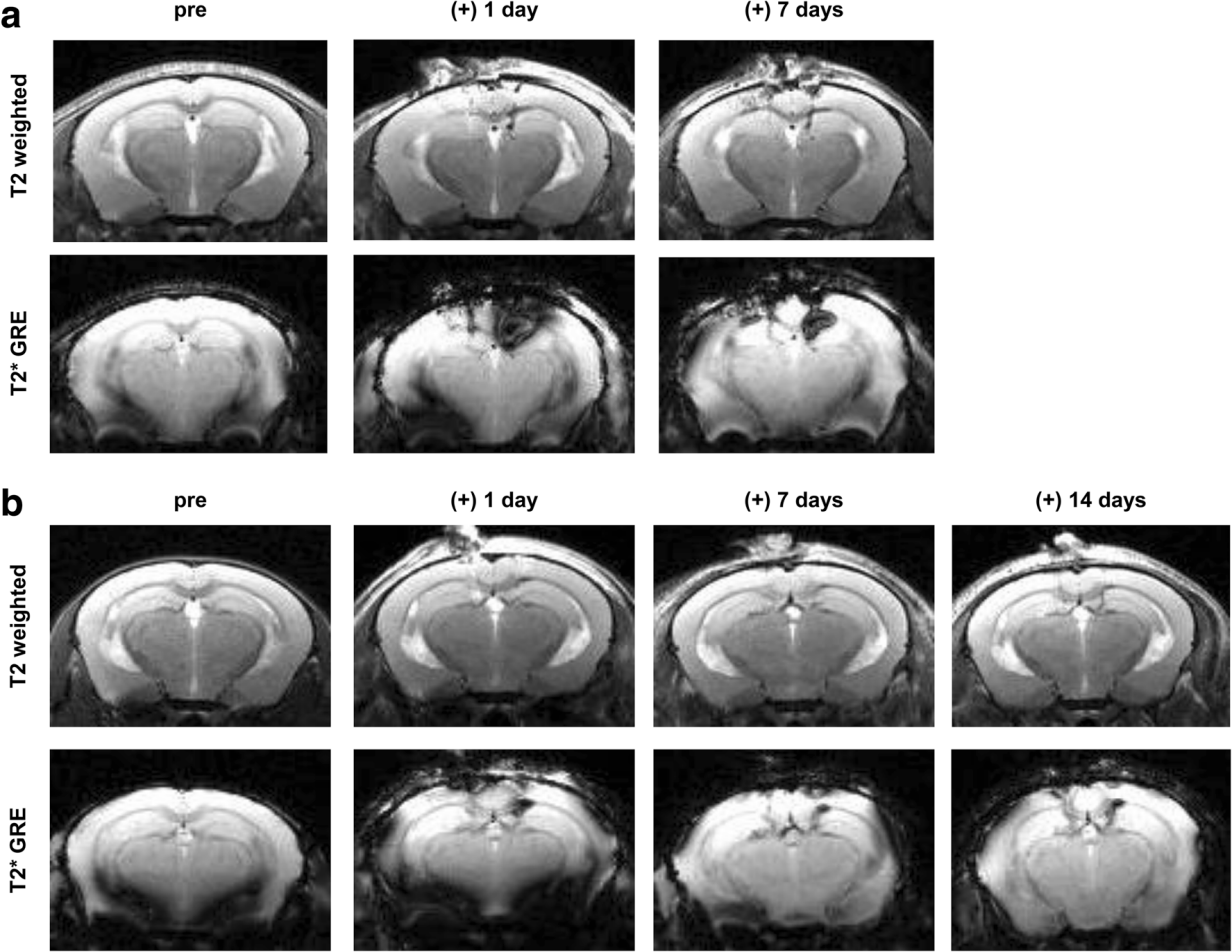
Hypointense Signals Consistently Visualized at Extended Time Points. MR images acquired up to (**a**) 7 and (**b**) 14 days post transplantation. Similar to early time points, hypointense signals were observed in the right hippocampus where the ferumoxtyol-labeled hUCB-MSCs were transplanted. A gradual decrease in the size of the signal intensity was apparent at post 7 days while striking differences were not noted between post 7 and 14 days

With such observations made at post 1 day, a question that arose was whether hypointense signals will be observed at the engraftment site past 1 day. Therefore, additional experiments were conducted under the same experimental settings as the post 1 day group, and the mice were monitored at 2 time points: post 7 and 14 days. For both groups, at both post 7 days, the hypointense signal was consistently observed at the right hippocampus (Fig. 7a, b). Especially discernible from the T2* GRE images, the overall size of the signal was decreased, when compared to the image acquired at post 1 day (Fig. 7a, b). The size and appearance of the signal detected at post 14 days resembled that observed at post 7 days (Fig. 7b).

## Discussion

In this study, ferumoxytol-labeled hUCB-MSCs that were engrafted in the hippocampus of 5XFAD transgenic AD mice were observed using MRI with the hope of supporting the application of this technique in AD stem cell therapy. Although invasive brain penetration and confined cellular distribution are disadvantages of stereotactic delivery, the method is still a well-established technique that has been widely used in small animals and allows for localization of cells at the target site [40]. In our study, we targeted the hippocampi of 5XFAD transgenic mice that are known to develop high amyloid deposition at very early stages of disease progression [41]. Considering these pathological features and the presence of the blood brain barrier (BBB), intra-parenchymal administration is preferred in the clinical settings; our group has clinically confirmed the safety and feasibility of delivering stem cells by utilizing this route in AD patients [22].

The first goal of this study was to demonstrate the feasibility of labeling hUCB-MSCs with ferumoxytol. Our results were in agreement with past studies that supported the use of the heparin-protamine sulfate-ferumoxytol nanocomplex as an efficient labeling method [29, 30, 34]. We observed *in vitro* ferumoxytol labeling of hUCB-MSCs in both adhered and detached states. Iron-positive staining of detached ferumoxytol-labeled hUCB-MSCs confirmed the stability of ferumoxytol labeling even after multiple washings, centrifugation, and re-suspension. In addition to efficient labeling, ferumoxytol labeling did not negatively influence the expression of certain cell surface markers or the differentiation capabilities of hUCB-MSCs *in vitro*. The fibroblastic characteristics and overall proliferation of the labeled cells were not impaired [42, 43]. There are evidences of MSCs differentiating into ectodermal lineages if pre-induced or introduced to developmental cues in vivo [44, 45], but examining such potentials was not defined as the major objectives of this present study.

The second goal of this study was to observe the engraftment of ferumoxytol-labeled hUCB-MSCs at the target site. As expected, iron-positive hUCB-MSCs were accurately delivered and engrafted into the mouse hippocampus. Growth of the transplanted cells was not examined in depth because MSCs have a low probability of proliferating in vivo, which is probably why they do not generate teratomas unlike embryonic and induced pluripotent stem cells [46, 47]. Considering the aggregated state of the cells, limited distribution seem to have occurred for both unlabeled and ferumoxytol-labeled hUCB-MSCs. Compared to the left hippocampus (unlabeled hUCB-MSCs as a control), a prominent negative contrast was discernible from the right hippocampus where ferumoxytol-labeled hUCB-MSCs were injected. Such hypointense signals were visualized at various time points from immediately to post 14 days following stereotactic delivery. In contrast to the T2-weighted images, the enlarged areas of dark signals in the T2* GRE images are representative of the blooming effect or susceptibility artifacts which decreased with time [48]. While T2 weighted spin echo lacks iron sensitivity, T2* is much more sensitive but does not provide a reliable representation of the engraftment size. Thus, the two sequences compensate for each other’s weaknesses and were both used to noninvasively image the engraftment site.

Other than the blooming effect, alternative confounding factors that may restrict the interpretation of MR images include (but are not limited to) brain puncture, hemorrhage, and activation of macrophages. First, signs of hemorrhages were not recognized from MR images, which excluded bleeding as a source of the negative signal. Based on the images of the sham group, the hypointense signal was generated from penetration of the needle into the brain parenchyma and clearly differed from that created from the injection of ferumoxytol-labeled hUCB-MSCs [49, 50]. An interesting observation was that infiltration of inflammatory cells was not detected at the sites of engraftment for either unlabeled or ferumoxytol-labeled hUCB-MSCs. This indicated that macrophage engulfment of iron and/or dead cells were not the origin of the hypointensity [51]. However, former groups have proposed that SPIO-labeled cells underwent apoptosis and phagocytosis by macrophages when observed as early as 1 to 2 weeks post transplantation [51–54]. Considering such reports, we cannot rule out the possibility that apoptosis and inflammatory cell infiltration might be apparent as was as reduced cell survival at the site of ferumoxytol-labeled hUCB-MSC engraftment if monitored at later time points past 1 day.

This study has several limitations that must be discussed. First, as stated above, cell fate was not evaluated at time points past one day. However the gradual decrease in hypointensity observed over time is suggested to be indicative of cell survival [26]. Thus, we expect the residual, viable cells from the initial transplantation to contribute to the generation of the hypointense signal. Second, the efficacy of the ferumoxytol-labeled hUCB-MSCs in the transgenic AD mouse model was not examined in depth. The major aims of our study were to assess the feasibility of labeling hUCB-MSCs with ferumoxytol and their detection using MRI. As a result, potential therapeutic benefits or biological roles of ferumoxytol-labeled hUCB-MSCs in the host environment were not extensively investigated. Since the ferumoxytol-labeled hUCB-MSCs met the criteria proposed by the ISCT, we presume that the labeled cells would not fall short of the stemness, including immunophenotype and therapeutic potential, displayed by unlabeled, naïve MSCs. Further study is warranted to evaluate the efficacy of ferumoxytol-labeled hUCB-MSCs, their survival, and its correlation to the persistence of negative signals detected from MRI.

In summary, the results from the current study introduce use of the heparin-protamine sulfate-ferumoxytol nanocomplex to label hUCB-MSCs and show that labeled cells can be detected noninvasively using MRI following transplantation. We hope that this method will be applied as a follow-up for clinical cell transplantations not only in AD, but also in various other diseases.

## Electronic supplementary material

Supplementary Fig. 1 In vitro Cellular MRI of Ferumoxytol-Labeled hUCB-MSCs Suspended in Agarose. (A) Axial and sagittal images of phantom samples made by mixing unlabeled or ferumoxytol-labeled hUCB-MSCs with agarose. (B) Compared with the agarose and unlabeled hUCB-MSC samples, a reduction in signal intensity was observed from ferumoxytol-labeled hUCB-MSCs (average of 3 independent experiments, *P ≤ 0.05, **P ≤ 0.01). (GIF 10 kb)

High resolution image (TIFF 776 kb)

Supplementary Fig. 2 Immunophenotype Characterization of Ferumoxytol-Labeled hUCB-MSCs Suspended in Agarose. (A) Unlabeled and (B) ferumoxytol-labeled hUCB-MSCs were positive for antigens CD105, CD90, and CD73. Both cell types were negative for antigens HLA-DR, CD45, and CD14. (GIF 16 kb)

High resolution image (TIFF 1402 kb)
