# Supplementary figures and images for: Magnetic Resonance Imaging of Ferumoxytol-Labeled Human Mesenchymal Stem Cells in the Mouse Brain
Source: Stem Cell Rev. 2016 Oct 18;13(1):127–38. doi: 10.1007/s12015-016-9694-0 (PMC5346117; doi:10.1007/s12015-016-9694-0)

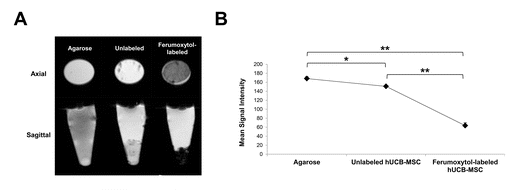

Supplement: Supplementary file 1 — In vitro Cellular MRI of Ferumoxytol-Labeled hUCB-MSCs Suspended in Agarose. (A) Axial and sagittal images of phantom samples made by mixing unlabeled or ferumoxytol-labeled hUCB-MSCs with agarose. (B) Compared with the agarose and unlabeled hUCB-MSC samples, a reduction in signal intensity was observed from ferumoxytol-labeled hUCB-MSCs (average of 3 independent experiments, *P ≤ 0.05, **P ≤ 0.01). (GIF 10 kb) [file 12015_2016_9694_Fig8_ESM.gif]

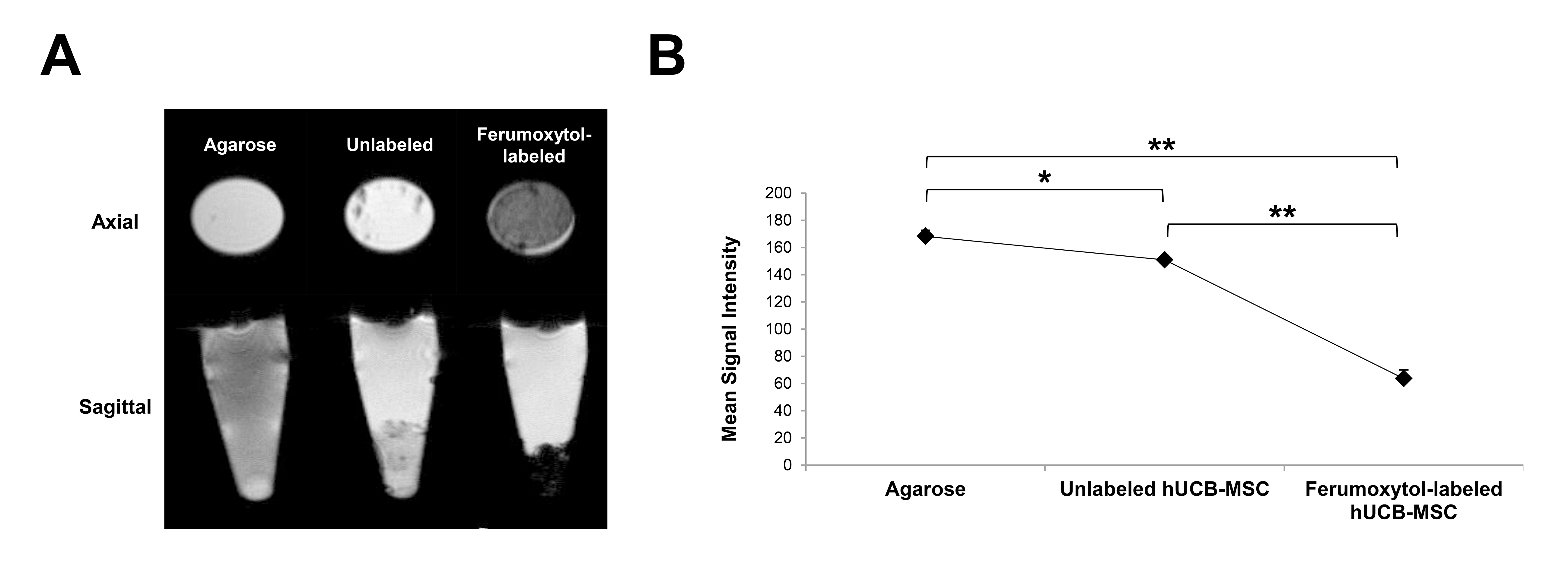

Supplement: Supplementary file 2 — High resolution image (TIFF 776 kb) [file 12015_2016_9694_MOESM1_ESM.tif]

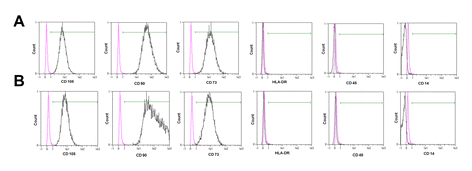

Supplement: Supplementary file 3 — Immunophenotype Characterization of Ferumoxytol-Labeled hUCB-MSCs Suspended in Agarose. (A) Unlabeled and (B) ferumoxytol-labeled hUCB-MSCs were positive for antigens CD105, CD90, and CD73. Both cell types were negative for antigens HLA-DR, CD45, and CD14. (GIF 16 kb) [file 12015_2016_9694_Fig9_ESM.gif]

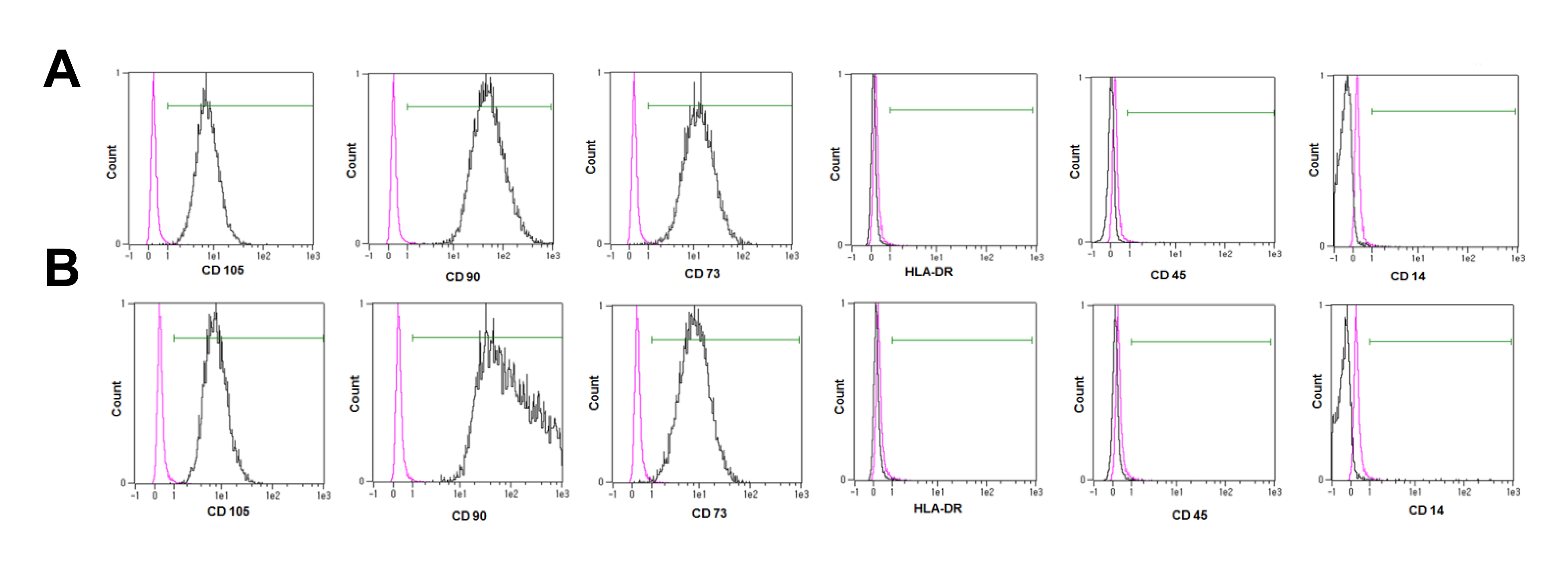

Supplement: Supplementary file 4 — High resolution image (TIFF 1402 kb) [file 12015_2016_9694_MOESM2_ESM.tif]
